# Pain, anxiety, and catastrophizing among pregnant women with dental pain, undergoing root canal treatment

**DOI:** 10.1590/1807-3107bor-2024.vol38.0054

**Published:** 2024-06-24

**Authors:** Andressa Heberle GASTMANN, Samantha Rodrigues XAVIER, Katerine Jahnecke PILOWNIC, Ana Regina ROMANO, Fábio de Almeida GOMES, Marília Leão GOETTEMS, Renata Dornelles MORGENTAL, Fernanda Geraldo PAPPEN

**Affiliations:** (a) Universidade Federal de Pelotas – UFPel, School of Dentistry, Graduate Program in Dentistry, Universidade Federal de Pelotas, Pelotas, RS, Brazil; (b) Universidade de Fortaleza – Unifor, Faculty of Dentistry, Department of Endodontics, Fortaleza, CE, Brazil.; (c) Universidade Federal de Santa Maria – UFSM, School of Dentistry, Department of Stomatology, Santa Maria, RS, Brazil.

**Keywords:** Catastrophization, Dental Anxiety, Pregnancy

## Abstract

This prospective study aims to evaluate dental pain, anxiety, and catastrophizing levels in pregnant women undergoing root canal treatment. Sixty pregnant and non-pregnant women presenting dental pain and an indication for root canal treatment were included in the study. Dental anxiety and catastrophizing were investigated using validated questionnaires. The endodontic intervention was performed, and a numerical scale measured preoperative and postoperative dental pain. The results were analyzed using STATA software 12.0. Unadjusted analyses assessed the association between pregnancy and pain, anxiety, and catastrophizing levels. Multiple linear regression models using ‘forward stepwise’ entry procedures were used to assess the independent effects of variables on pain scores. The significance level was set at 0.05. Initially, most patients experienced intense dental pain. The levels of dental pain, dental anxiety, and catastrophizing did not differ between pregnant and non-pregnant women. Logistic regression showed that postoperative pain was associated with irreversible pulpitis diagnosis (OR = 4.78; 95%CI 1.55–13.55) and high catastrophizing levels (OR = 1.96; 95%CI 1.01–3.84). Preoperative and postoperative pain rates and anxiety and catastrophizing were similar between pregnant and non-pregnant patients. Postoperative pain was associated with irreversible pulpitis diagnosis and high catastrophizing levels. The similarity between pregnant and non-pregnant women regarding preoperative and postoperative dental pain and catastrophizing and anxiety levels supports the indication of root canal treatment during the gestational period whenever necessary.

## Introduction

The oral health of pregnant women is an issue whose importance and impact on pregnancy outcomes is recognized by obstetricians.^1^ The physiological changes in pregnancy include changes in the oral cavity and increased susceptibility to oral infections.^2^ Increased consumption of carbohydrates, vomiting, increased acidity in the oral cavity, and the reduced production of saliva have been reported as factors responsible for the greater risk of dental caries during pregnancy.^3,4^ Moreover, pregnant women are more susceptible to gingivitis, dental mobility, dental caries, and erosion.^5-7^ Survey data indicate that up to 54.9% of pregnant women seek out dental care during pregnancy with acute dental pain as the main motivation;^8,9^ consequently, emergency dental care for acute dental pain are frequent in this period.^8^

Dentists frequently have to deal with the anxiety associated with the safety of dental treatment during pregnancy, while at the same time having to eliminate odontogenic infections. Consequently, many dentists are cautious when treating pregnant women,^10,11^ while obstetricians do not usually recommend prenatal dentistry to their patients.^12^ Due to misconceptions and lack of information, pregnant women frequently receive a prescription and/or over-the-counter analgesics to control severe pulpal pain. Abuse of these drugs rather than appropriate dental treatment may have deleterious effects on the fetus and the pregnant woman.^1^ Therefore, it is imperative that odontogenic infections be treated promptly at any time during pregnancy.^13^ There seems to be no reason to prevent pregnant women from receiving dental treatment during pregnancy if all the recommendations for radiographic exposure^14^ and local anesthesia^7^ are followed.

The limitations and barriers of patients and healthcare professionals have a substantial effect on dental treatment during pregnancy. It has been reported that pregnant women do not seek dental treatment due to fear and apprehension, and lack of awareness about dental problems and misconceptions about the effect of dental treatment on fetal development.^15^ Thus, it is possible that pregnant patients are more anxious about dental treatment than non-pregnant women, especially if they have dental pain. Dental appointments can cause stress, fear, and anxiety, and these feelings are intensified by the perception of pain that the anxious or fearful person may be experiencing. Moreover, patients with symptoms related to negative and catastrophic thoughts may be more likely to suffer emotionally and have longer symptoms. Although catastrophizing has not been reported to be influenced by hormonal changes, and even no sex difference in pain catastrophizing has been described^16,17^, catastrophizing has risen to the status of one of the most important psychological predictors of the pain experience and can be defined overall “as an exaggerated negative reaction to actual or anticipated pain experience”.^18^

Considering that anxiety and catastrophic thoughts may be amplified in pregnant women experiencing dental pain, this study aimed to assess the perception of pain during root canal treatment, dental anxiety, and catastrophic thinking in pregnant women and non-pregnant patients.

## Methodology

### Ethical Considerations

This study was undertaken with the approval of the Institutional Research Ethics Committee from the Federal University of Pelotas, Brazil (CAAE 50753815.6.0000.5318). The patients were screened by one clinician who also obtained the consent from patients enrolled in the study.

All participants were informed of the procedure protocols, risks and benefits, and their right to interrupt their participation. Written consent was signed, and a copy was delivered to all volunteers.

### Sample and study design

A cross-sectional study was conducted, and patients seeking emergency dental care with irreversible pulpitis or necrotic pulp with acute apical periodontitis/abscess were recruited. This study is reported according to the STROBE guideline for cross-sectional studies (www.strobe-statement.org). The sample size calculation considered the primary and secondary outcomes. For catastrophizing prevalence, the catastrophic pain scale (PCS) for pregnant (26 SD 9)^19^ and non-pregnant women (20.14 SD 9)^20^ were considered. Also, a 76.7% prevalence of dental anxiety among pregnant women^21^ and a 40.9% prevalence of dental anxiety among non-pregnant women^22^ were considered. Finally, a 54.9% prevalence of dental pain among pregnant women^9^ and a 19.2% prevalence of dental pain in non-pregnant women (control group)^23^ were used. A type I error of 5% and a power of 80% were assumed. The calculation showed that a minimum of 28 subjects were required in each group for this study. Considering the possibility of information loss, the sample size was increased by 10%.

### Patient selection

Patients were screened between June 2018 and December 2019 at the Faculty of Dentistry of Pelotas. The inclusion criteria were females older than 16 years and younger than 40, with spontaneous dental pain at the moment of dental intervention or in the previous 24 hours, and clinical diagnosis of symptomatic irreversible pulpitis or necrotic pulp with acute apical periodontitis or abscess due to caries. Exclusion criteria were patients with pain from other dental origins, dental pain due to traumatic injuries, and patients with systemic diseases that could interfere with the immunological response. The diagnosis was based on pain history, lingering pain following thermal testing, and radiographic examination. To be allocated to the pregnant group, the women had to present a positive quantitative blood test [serum beta], while non-pregnant women had to confirm the regularity of their menstrual period to be included in the study.

Two previously trained graduate dental students collected the data and performed all the endodontic procedures.

### Measures

#### Pain scale

Each participant was invited to record their pain experience score using a Numerical Rating Scale (NRS) at two different moments: when arriving at the dental appointment and 24 hours after the root canal treatment. The NRS is an 11-point scale ranging from 0 to 10, in which 0 represents ‘’no pain’’ and 10 represents “the worst pain that one can imagine”. The scale was self-administered and respondents were asked to place a vertical mark on the scale where it best represented their pain intensity at that moment.^24^. Pain was later categorized into mild (1–3), moderate (4–6), and severe (7–10) to facilitate clinical interpretability.

#### Dental anxiety

To evaluate dental anxiety, a questionnaire modified from the original version of the Dental Anxiety Scale (DAS) and validated in Portuguese, was applied (DAS-R).^25^ The scale contains four multiple-choice questions that assess the degree to which participants experience fear or anxiety when thinking of different aspects of dental procedures (i.e. preparing for a check-up, waiting for their turn in the chair, waiting while the dentist prepares the drill, and waiting while the dentist or hygienist prepares the scaling instruments). Each item is scored on a scale of 1 to 5, ranging from calm (score 1) to terrified (score 5). The sum of scores for all items resulted in values from 4 to 20.^25^ Values above 15 were categorized as extreme dental anxiety, values between 12 and 14 as moderate anxiety, and below 11 as low anxiety.

#### Catastrophizing

A version of the PCS translated to Portuguese was used to evaluate patients’ catastrophic thinking.^26^ The PCS is a 13-item self-report measure with scores ranging from 0 (no catastrophizing) to 52 (severe catastrophizing). Respondents rate how frequently they experience different thoughts and feelings when in pain. Ratings are made on a 5-point scale with the endpoints of 0, not at all, 1, to a slight degree, 2, to a moderate degree, 3, to a great degree, and 4, all the time.^27^ The PCS yields a total score and three dimensions of pain catastrophizing: magnification (three items, *i.e.* “I wonder whether something serious may happen”), rumination (four items; *i.e.* “I keep thinking about how much it hurts”), and helplessness (six items; *i.e.* “I cannot reduce the intensity of the pain”).^26^ For statistical analysis, the PCS variable was categorized using the median value as a reference. Patients scoring 37 or higher were considered to have high pain catastrophizing, whereas patients who scored less than 37 cut-off point were considered to have low pain catastrophizing.

#### Endodontic Procedures

For maxillary teeth, local anesthesia was achieved by buccal infiltration with 3.6 mL of lidocaine with 1:100,000 epinephrine (Alphacaine, Nova DFL, Jacarepaguá, RJ, Brazil). For mandibular teeth, a dental nerve block was usually performed. A dose limit of 3.6 mL was considered for each session. A rubber dam was placed and disinfected with sodium hypochlorite, carious lesions were excavated and the pulp chamber was accessed using sterile carbide burs. A glide path was established with stainless steel hand instruments up to size #15. The canal was instrumented with nickel-titanium rotary instruments (ProTaper Next; Dentsply Maillefer, Ballaigues, Switzerland). The working length of the apical constriction was electronically verified (Romidan Ltd, Kiryat Ono, Israel). The root canal filling was performed using the single cone technique with AH Plus^®^ sealer (De Trey-Dentsply, Konstanz, Germany). The teeth were restored with a direct adhesive build-up with a composite resin material.

#### Statistical Analysis

Independent double-entry verification was performed to ensure the accuracy of dates. The Software STATA version 12.0 was used for the analyses. Simple descriptive statistics were generated. Unadjusted analyses were undertaken using the chi-square test to assess the associations between pain scores and the independent variables according to the groups (pregnant or non-pregnant women). Multiple linear regression models using ‘forward stepwise’ entry procedures were used to assess the independent effects of variables on pain scores. The significance level was set at 0.05.

## Results

The frequency distribution for some of the sample characteristics and their association with pregnancy are presented in Table 1. Initially, a total of 62 participants were included in the study. Two participants did not complete the root canal treatment and were excluded from further statistical analysis. Sixty women, 30 (50.0%) non-pregnant and 30 (50.0%) pregnant women with a mean age of 26.6 (SD 5.8) years were included in the statistical analysis. Among pregnant participants, 5 (16.7%) were up to 12 weeks pregnant, 12 (40.0%) were 13 to 24 weeks pregnant, and 13 (43.3%) were 25 weeks pregnant or more. Most participants had no previous root canal treatment (68.3%) and the mean pain rating was 8.02 (SD 1.97), indicating that most subjects experienced intense dental pain. The anxiety levels among participants had decreased from the first appointment (10.48, SD 3.94) in comparison to the levels obtained after root canal instrumentation (1.20, SD 1.35) and after root canal filling (0.78, SD0.92).

**Table 1:** Sample characteristics (n=60) of pregnant and non-pregnant women.

Variables	Non-pregnant	Pregnant	P-value*

	30 (50%)	30 (50%)	
Age			0.121
16 – 25 years	11 (39.3)	17 (60.7)	
26 – 40 years	19 (59.4)	13 (40.6)	
Education level			0.426
≤ 8 years	10 (43.5)	13 (56.5)	
> 8 years	20 (54.1)	17 (45.9)	
Family income (minimum wages)			
≥ 3	10 (33.3)	9 (30)	0.000
From 1.1 to 3	14 (46.7)	17 (56.7)	
≤ 1	6 (20)	4 (13.33)	
Marital status			
Married	25 (83.3)	26 (86.7)	0.000
Single	4 (13.3)	3 (10)	
Separated or divorced	1 (3.3)	1 (3.3)	
Previous root canal treatment			0.781
No	21 (51.2)	20 (48.8)	
Yes	9 (47.4)	10 (52.6)	
Diagnosis			0.795
Irreversible pulpitis	17 (51.5)	16 (48.5)	
Necrotic pulp with acute apical periodontitis/abscess	13 (48.1)	14 (51.9)	
Tooth			0.605
Anterior	4 (36.4)	7 (63.6)	
Pre-molar	7 (53.7)	6 (46.2)	
Molar	19 (52.8)	17 (47.2)	
Number of root canals			1.000
1-2	13 (50.0)	13 (50.0)	
3-4	17 (50.0)	17 (50.0)	

*Pearson Chi-square.

Table 2 describes the levels of preoperative and postoperative pain, dental anxiety, and catastrophizing among the study participants. The reported dental pain reduced significantly after the dental intervention (p < 0.001). Pregnant and non-pregnant women did not differ significantly for preoperative pain (p = 0.836) and postoperative pain ratings (p = 0.770). In addition, dental anxiety and catastrophizing levels were not different among pregnant and non-pregnant women (p > 0.05).

**Table 2:** Association between pregnancy and levels of dental pain, anxiety, and catastrophizing

Independent variable: Pregnancy				

Dependent variables	Total	Non-pregnant	Pregnant	P-value
Dental anxiety				0.866
Low	36 (60)	17 (56.7)	19 (63.3)	
Moderate	15 (25)	8 (26.7)	7 (23.3)	
Severe	9 (15)	5 (16.7)	4 (13.3)	
Catastrophizing levels				1.000
Low	30 (50)	15 (50)	15 (50)	
High	30 (50)	15 (50)	15 (50)	
Initial pain				0.836
Mild	3 (5)	1 (3.3)	2 (6.7)	
Moderate	16 (26.7)	8 (26.7)	8 (26.7)	
Severe	41 (68.3)	21 (70)	20 (66.7)	
Post-operative pain				0.779
Mild	41 (68.3)	21 (70)	20 (66.7)	
Moderate	12 (20)	5 (16.7)	7 (23.3)	
Severe	7 (11.7)	4 (13.3)	3 (10)	

Data are reported as number and percentage.

Considering the dental anxiety levels, 15.0% of the participants had scores suggestive of extreme dental anxiety (DAS range 15–20), 25.0% of moderate anxiety (DAS range 12–14), and 60.0%% of low dental anxiety (DAS < 11). The mean DAS-R was 10.48 (SD 3.94) and there was no association between the DAS-R and pregnancy (p = 0.866).

Anxiety and catastrophizing scores with preoperative and postoperative pain among pregnant and non-pregnant women were also assessed. The frequency of each score is described in Table 3.

**Table 3:** Catastrophizing, anxiety, and pain levels within the studied population

	Initial pain (%)			Post-operative pain (%)		

	Mild	Moderate	Severe	Mild	Moderate	Severe
Non-pregnant						
Low catastrophizing	1(100)	4(50)	10(47.6)	12(54.5)	1(25)	2(50)
High Catastrophizing	0(0)	4(50)	11(52.4)	10(45.5)	3(75)	2(50)
Low anxiety	1(100)	6(75)	10(47.6)	12(54.5)	3(75)	2(50)
Moderate anxiety	0(0)	1(12.5)	7(33.3)	7(31.8)	1(25)	0(0)
Extreme anxiety	0(0)	1(12.5)	4(19)	3(13.6)	0(0)	2(50)
Pregnant woman						
Low catastrophizing	1(50)	4(50)	10(50)	11(55)	3(42.9)	1(33.3)
High Catastrophizing	1(50)	4(50)	10(50)	9(45)	4(57.1)	2(66.7)
Low anxiety	0(100)	6(75)	13(65)	11(55)	5(71.4)	3(100)
Moderate anxiety	2(0)	1(12.5)	4(20)	6(30)	1(14.3)	0(0)
Extreme anxiety	0(0)	1(12.5)	3(15)	3(15)	1(14.3)	0(0)

Crude analysis revealed that postoperative pain was associated with the diagnosis of irreversible pulpitis (OR = 4.36; 95%CI: 1.41–13.55). After adjustments, the postoperative pain remained associated with irreversible pulpitis diagnosis (OR = 4.78; 95%CI: 1.55–13.55) and high catastrophizing levels (OR: 1.96; 95%CI: 1.01–3.84) (Table 4).

**Table 4:** Logistic regression of factors associated with dental pain

	Post-operative pain	P-value	Post-operative pain	P-value
	
	Crude		Adjusted*	
	
	OR (95% IC)		OR (95% IC)	
Group		0.783		0.651
Non-pregnant	1.00		1.00	
Pregnant	1.11 (0.52-2.36)		1.16 (0.60-2.27)	
Dental anxiety		0.617		0.392
Low	1.00		1.00	
Moderate/high	0.55 (0.18-1.68)		0.71 (0.33-1.55)	
Catastrophizing		0.414		0.049
Low	1.00		1.00	
High	1.38 (0.64-2.95)		1.96 (1.01-3.84)	
Diagnosis		0.011		0.007
Necrotic pulp	1.00		1.00	
Irreversible pulpitis	4.36 (1.41-13.55)		4.78 (1.55-14.75)	
Tooth		0.137		0.813
Anterior	1.00		1.00	
Pre-molar	4.23 (0.57-31.51)		1.34 (0.14-12.56)	
Molar	3.97 (0.57-27.50)		1.37 (0.15-12.39)	

## Discussion

To the best of our knowledge, this clinical study is the first one to assess dental pain correlated to dental anxiety and catastrophic thinking in pregnant women submitted to root canal treatment. The collected data showed that pregnant women did not differ from non-pregnant women regarding levels of preoperative and postoperative pain. There were also no differences between pregnant women and the control group concerning catastrophizing and dental anxiety, as could be expected. This topic is particularly interesting considering that the need for oral health care in pregnancy is higher,^6,7^ mainly due to the increased incidence and severity of oral inflammation during pregnancy.^28^ In addition, acute dental pain episodes make emergency dental treatment often necessary during this period.^8,9^ Contradictorily, few pregnant patients seek dental treatment, due to the anxiety that exists about dental intervention and misconceptions about the effect of treatment on fetal development.^15^

Dental pain should be eliminated regardless of the trimester of pregnancy.^29^ Due to persistent taboos and myths, it is not rare that dentists perform only emergency care for pain management.^30^ These myths, both by the pregnant woman and by the health professionals themselves, are the greatest barrier to dental treatment during pregnancy.^31^ The most common misconception that leads to care being postponed includes the exposure to X-rays, the prescription of analgesics and antibiotics, and the use of local anesthesia.^31^ However, there is no data to suggest that dental treatments and the use of local anesthetics during pregnancy are associated with an increased risk of abnormalities in the baby.^13^

The use of pretreatment dental anxiety questionnaires may help identify patients with fear and anxiety and may help in patients’ management and treatment planning. To achieve its purpose, the present study applied methods widely used in other investigations. The NRS, which measures preoperative and postoperative dental pain, is among the most commonly used tools to access pain intensity in clinical scenarios and has been previously validated in the Portuguese language^32^. In addition to its cross-cultural reliability, the NRS is slightly more sensitive than other measures and might be considered when particularly sensitive and responsive measures of pain intensity are needed.^32^ Furthermore, since catastrophizing has been described as a predictor of depression and anxiety,^33^ it is warranted to examine this characteristic in the studied population.

Our results indicated that most participants had intense dental pain, which significantly reduced after the dental intervention. Analogous results were observed in a systematic review conducted by Pak & White,^34^ in which pain incidence in the first 24 hours was 40%, decreasing acutely thereafter, and reaching incidence levels of 11% at 7 days. Likewise, pain severity declined substantially soon after treatment. Pregnant and non-pregnant women did not differ significantly in preoperative and postoperative pain ratings. It is important to highlight that age, education level, number of root canals, and diagnosis were similarly distributed among pregnant and non-pregnant women, and thus did not interfere with these results.

Among the participants of our study, 60.0% had low dental anxiety, corroborating the findings reported by Goettems et al.,^22^ who described that 59.5% of women displayed low dental fear, 18.1% had moderate dental fear, and 22.4% had high dental fear. In contrast, considering only pregnant women, Meneses et al.^21^ described a prevalence of 41.4% with extreme dental anxiety, while we found a prevalence of less than 16% of pregnant women with severe levels of dental anxiety.

The DAS-R and B-PCS rates were not different among pregnant and non-pregnant women as could be predicted. These results may be due to the fact that pregnant women with higher levels of anxiety are more likely to avoid dental appointments, preventing participation in the present study. Additionally, our intervention was performed in a program specialized in the attendance of pregnant women and their babies by dentists who routinely treat expectant mothers. Thus, the way that these patients were assisted might have interfered with the results, since before each appointment, the importance of appropriate treatment of dental pain and infection was emphasized. It has also been advocated that root canal treatment, when indicated, should not be deferred until after delivery to avoid the inappropriate long-term use of analgesics, and that root canal treatment has been considered safe during the gestational period.^13^

The anxiety levels decreased in the second dental appointment in comparison to the first appointment for both pregnant and non-pregnant patients. Probably, the reduction of pain after the dental intervention contributed to these findings. Similarly, two recent systematic reviews also described a substantial decrease in anxiety from the pretreatment stage to the treatment stage.^35,36^

In the present study, postoperative pain remained associated with high catastrophizing levels (OR 1.96). According to previous studies, individuals who catastrophize experience more pain and emotional distress during aversive or painful procedures than individuals who do not catastrophize.^26,37^ Also, a recent systematic review confirmed that dental anxiety (DA), as a dispositional factor toward the dental situation, is associated with pain related to dental procedures.^36^ However, our results did not confirm this assumption, since our data demonstrated that anxiety levels were not associated with higher levels of pain.

Logistic regression also demonstrated that teeth with vital pulp were associated with a higher incidence of postoperative pain in comparison to teeth with pulp necrosis. Our findings agree with those of Udoye and Jafarzadeh^38^ and Gotler et al.^39^ who also reported a higher frequency of postoperative pain following treatment of teeth with vital pulp. According to Gotler et al.,^39^ the cause of the higher incidence of postoperative pain in teeth with vital pulp may be the injury to the periapical vital tissue during root canal instrumentation and irrigation, which in teeth with vital pulp promotes more severe exudation of inflammatory mediators, such as prostaglandins, leukotrienes, serotonin, histamine, and bradykinin, which are pain mediators.

From the clinical perspective, our findings imply that catastrophizing and anxiety assessment can be useful in the management of patients presenting with extreme anxiety. Most of these tools are short in length, and the patients themselves can easily complete them. These assessments could be included as part of the initial evaluation of the patient’s condition, and their ratings at different treatment stages could provide critical information for dentists in the evaluation of treatment outcomes. In addition, the similarity between pregnant and non-pregnant women regarding preoperative and postoperative dental pain and catastrophizing and anxiety levels confirms the indication of root canal treatment during the gestational period whenever it is necessary. Yet, further studies assessing the role of hormonal levels of pregnant women are required.

## Conclusions

Preoperative and postoperative pain, anxiety, and catastrophizing thoughts were similar among pregnant and non-pregnant patients. Postoperative pain was associated with irreversible pulpitis diagnosis and high catastrophizing levels.
